# mRNA transfection by a Xentry-protamine cell-penetrating peptide is enhanced by TLR antagonist E6446

**DOI:** 10.1371/journal.pone.0201464

**Published:** 2018-07-30

**Authors:** Glenn D. Bell, Yi Yang, Euphemia Leung, Geoffrey W. Krissansen

**Affiliations:** Department of Molecular Medicine & Pathology, Faculty of Medical and Health Sciences, The University of Auckland, Auckland, New Zealand; Universidad de Castilla-La Mancha, SPAIN

## Abstract

Messenger RNA (mRNA) transfection is a developing field that has applications in research and gene therapy. Potentially, mRNA transfection can be mediated efficiently by cell-penetrating peptides (CPPs) as they may be modified to target specific tissues. However, whilst CPPs are well-documented to transfect oligonucleotides and plasmids, mRNA transfection by CPPs has barely been explored. Here we report that peptides, including a truncated form of protamine and the same peptide fused to the CPP Xentry (Xentry-protamine; XP), can transfect mRNAs encoding reporter genes into human cells. Further, this transfection is enhanced by the anti-malarial chloroquine (CQ) and the toll-like receptor antagonist E6446 (6-[3-(pyrrolidin-1-yl)propoxy)-2-(4-(3-(pyrrolidin-1-yl)propoxy)phenyl]benzo[d]oxazole), with E6446 being >5-fold more potent than CQ at enhancing this transfection. Finally, E6446 facilitated the transfection by XP of mRNA encoding the cystic fibrosis transmembrane regulator, the protein mutated in cystic fibrosis. As such, these findings introduce E6446 as a novel transfection enhancer and may be of practical relevance to researchers seeking to improve the mRNA transfection efficiency of their preferred CPP.

## Introduction

Messenger RNA (mRNA) has potential advantages over DNA as an alternative for use in gene therapy [1–3]. For example, unlike DNA, mRNA cannot integrate into the genome, so there is no risk of insertional mutagenesis leading to oncogenesis. Further, mRNA only needs to reach the cytoplasm to be expressed, whereas DNA must be delivered into the nucleus [4]; thus DNA-based gene therapies are either limited to dividing cell populations, where nuclear envelopes break down during cell division, or require the use of inherently risky viral vectors. Additionally, mRNA transcripts are smaller and simpler to engineer than DNA, as there is no need for promoter and terminator sequences, and mRNA’s transient nature may allow improved control over protein expression kinetics. Together, these attributes could make gene therapy safer, cheaper, and quicker to enter into clinical testing [1–3].

However, gene therapy using mRNA faces one same major obstacle to success as gene therapy using DNA: simply, there is no safe and effective way to deliver genes into many epithelial and muscle tissues *in vivo* [5]. These tissues are affected by various disorders potentially amenable to gene therapy, including cystic fibrosis (CF)—the most common life-shortening monogenetic disorder [6]—the muscular dystrophies [7], and cardiovascular disease [8]. Current gene therapy vectors have drawbacks that preclude their use in targeting these tissues. More specifically, viral vectors are limited by their immunogenicity, the risk of insertional mutagenesis, and difficulties in production [9–12]; non-viral vectors are limited by their toxicity and low efficiency [13–16]; and both types of vector have limited ability to target specific tissues [11, 12, 17].

One method to mitigate the issue of tissue-specific targeting of gene therapy is topical application. However, this is not possible with many epithelial and muscle tissues and where it is possible various physical barriers to gene delivery present. For example, inhalation is a route for topical application of the lungs, a major target for gene therapy of CF. However, the mucus and alveolar fluid layers, which blanket respiratory epithelial cells, are barriers to the entry of foreign materials [18]. These layers contain secreted lipids, peptides, and proteins which may bind to gene therapy vectors and hinder their diffusion. Notably, the CF lung produces thicker mucus, thereby compounding this issue. Further, these fluid blankets, subject to the combined actions of beating cilia, function as a continuous conveyor belt transporting ensnared molecules out of the lungs [18]. Finally, the glycocalyx, the extracellular polysaccharide-rich coating underlying the fluid layers, acts as an additional impediment to gene delivery into lung epithelia [19].

A potential way to overcome the issue of gene delivery to such tissues is via the use of the cell-penetrating peptide (CPP) Xentry [20]. Xentry is a new class of CPP which can deliver macromolecules—such as siRNA, peptides, and proteins—into cells. Uniquely, Xentry enters adherent cells exclusively, whereas CPPs typically enter cells indiscriminately [21, 22]. Thus, intravenous administration of Xentry, to side-step the barriers which topical application presents, should not be hampered by blood cell sequestration. Further, Xentry’s utility may be extended by fusion to functional peptide sequences, such as those which bind and protect nucleic acids or home to specific tissues [20, 23]. Finally, Xentry’s cell-penetrating ability can be conditionally controlled by fusing Xentry to itself via a protease-cleavable peptide motif [24]. This twin-Xentry arrangement prevents cell entry until the protease-specific motif is cleaved, and the use of a motif cleavable by proteases expressed exclusively in the target tissue enables tissue-specific targeting.

Tissue-targeted delivery of exogenous mRNA may also need to be complemented, for optimal gene expression, by the modulation of cellular innate immune responses. Such responses may inhibit therapeutic protein production, through nuclease activation and suppression of translation, as well as cause cell death [25]. The immune responses are mediated by pattern recognition receptors (PRRs), such as toll-like receptors (TLRs), which identify molecules, such as exogenous nucleic acids, that are typically associated with pathogens. In accordance, PRRs are normally expressed by cells that interact with microbes, such as immune cells and mucosal epithelial cells [26, 27]. As such, reagents which suppress innate immune responses have been observed to enhance gene expression when added to *in vitro* gene deliveries (transfections). These reagents include the antimalarials chloroquine (CQ) and hydroxychloroquine (HCQ) [28].

Here, we attempt to use Xentry as a base to deliver mRNA into human epithelial cells. We fuse Xentry to a truncated form of human protamine—a peptide that binds, compacts, and stabilizes DNA in sperm—and test the Xentry protamine fusion peptide (XP) for its ability to transfect reporter gene mRNAs into cell lines *in vitro*. Next, we compare the ability of CQ, HCQ, and TLR antagonist 6-[3-(pyrrolidin-1-yl)propoxy)-2-(4-(3-(pyrrolidin-1-yl)propoxy)phenyl]benzo[d]oxazole (E6446) to enhance this transfection. Finally, we assess the ability of XP and E6446 to transfect mRNA encoding the cystic fibrosis transmembrane regulator (CFTR), the protein mutated in CF, into epithelial cells *in vitro*. These findings provide insight into mRNA transfection by CPP and introduce E6446 as a novel transfection enhancer.

## Results

### CPPs transfect mRNA

In preliminary testing of mRNA transfection by XP, in cancer cell lines, we made several observations. Firstly, we observed reporter gene expression at very low levels (S1 Fig). Secondly, we observed similar levels of reporter gene expression where we replaced XP with truncated human protamine (S2 Fig). While novel, transfection of mRNA by truncated human protamine is not surprising considering that other forms of protamine can transfect nucleic acids [29, 30]. Thirdly, we observed that the addition of CQ or HCQ to XP transfections improved reporter gene expression (S3 Fig). Unfortunately, the use of CQ or HCQ in gene therapy is precluded by their toxicity *in vivo* [31, 32]. As such, we reviewed the literature for clues to alternative agents to use to enhance mRNA transfection by XP. The review identified a promising candidate in the form of E6446, a small molecule which antagonizes nucleic acid-sensing TLRs more potently than CQ or HCQ and is less toxic than CQ when administered to mice [33–35]. Thus, we wondered whether E6446 could enhance mRNA transfections by XP better than CQ or HCQ and with less toxicity.

### mRNA transfection is enhanced by TLR antagonist E6446

**Fig 1:**
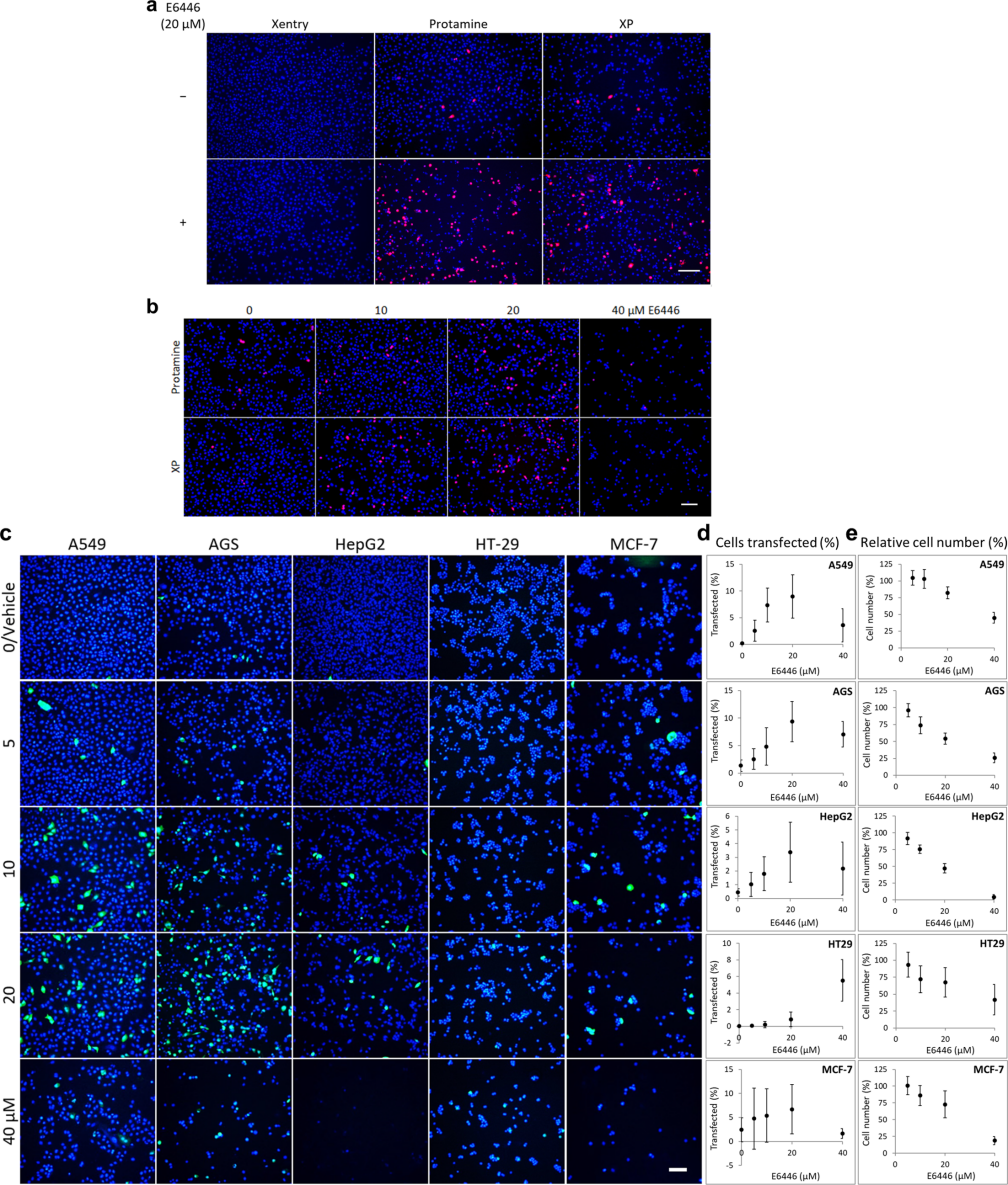
mRNA transfection by protamine-containing CPPs is enhanced by certain concentrations of TLR antagonist E6446. **(a)** Fluorescence imaging of RFP expression (red) in human gastric cancer (AGS) cells 24 h after treatment with RFP mRNA mixed with Xentry, truncated human protamine, or XP. **(b)** Fluorescence imaging of RFP expression (red) in AGS cells 24 h after treatment with RFP mRNA mixed with either truncated human protamine or XP. **(c, d)** Fluorescence imaging and quantification of EGFP expression (green) in human lung (A549), gastric (AGS), liver (HepG2), colon (HT-29), and breast (MCF7) cancer cell lines 24 h after treatment with EGFP mRNA mixed with XP and 0–40 μM E6446. Cell counts following transfection were also quantified **(e)**. Cell nuclei are stained blue with DAPI; scale bars = 100 μm. Data are shown as mean ± 95% confidence interval and are representative of 4+ independent experiments.

To test the ability of E6446 to enhance transfections, we treated cancer cell lines with CPPs that had been mixed with both E6446 and mRNA encoding fluorescent reporter genes. The cells were fixed 24 h after transfections, nuclear counterstained with DAPI, and then imaged by fluorescence microscopy. We found that 20 μM E6446 increased the number of gastric cancer (AGS) cells expressing red fluorescent protein (RFP) following mRNA transfections by either XP or truncated human protamine but not Xentry (Fig 1A). Further, 10 and 20 μM E6446 increased RFP expression in AGS cells following RFP mRNA transfection by XP or protamine, though 40 μM E6446 decreased both RFP expression and cell counts (Fig 1B). Finally, adding 5–20 μM E6446 to XP transfections of enhanced green fluorescent protein (EGFP) mRNA increased the percentage of cells expressing EGFP in five human cancer cell lines (2-way ANOVA: F_1,142_ = 12.80; *p <* 0.001; Fig 1C and 1D). More specifically, adding E6446 to XP increased EGFP expression from a baseline of 0–2% to 3–9% across the cell lines; with the addition of 20 μM E6446 producing the highest levels of expression for all cell lines except HT-29, where 40 μM was superior. Notably, though, E6446 concentrations were inversely associated with cell counts following transfection, with the addition of 20 μM E6446 reducing cell numbers by 18–53% across the cell lines (Fig 1E).

### E6446 is more potent than CQ at enhancing mRNA transfection and these reagents have additive effects on transfection

Next, we compared E6446 with CQ for its ability to enhance mRNA transfections by XP.

**Fig 2:**
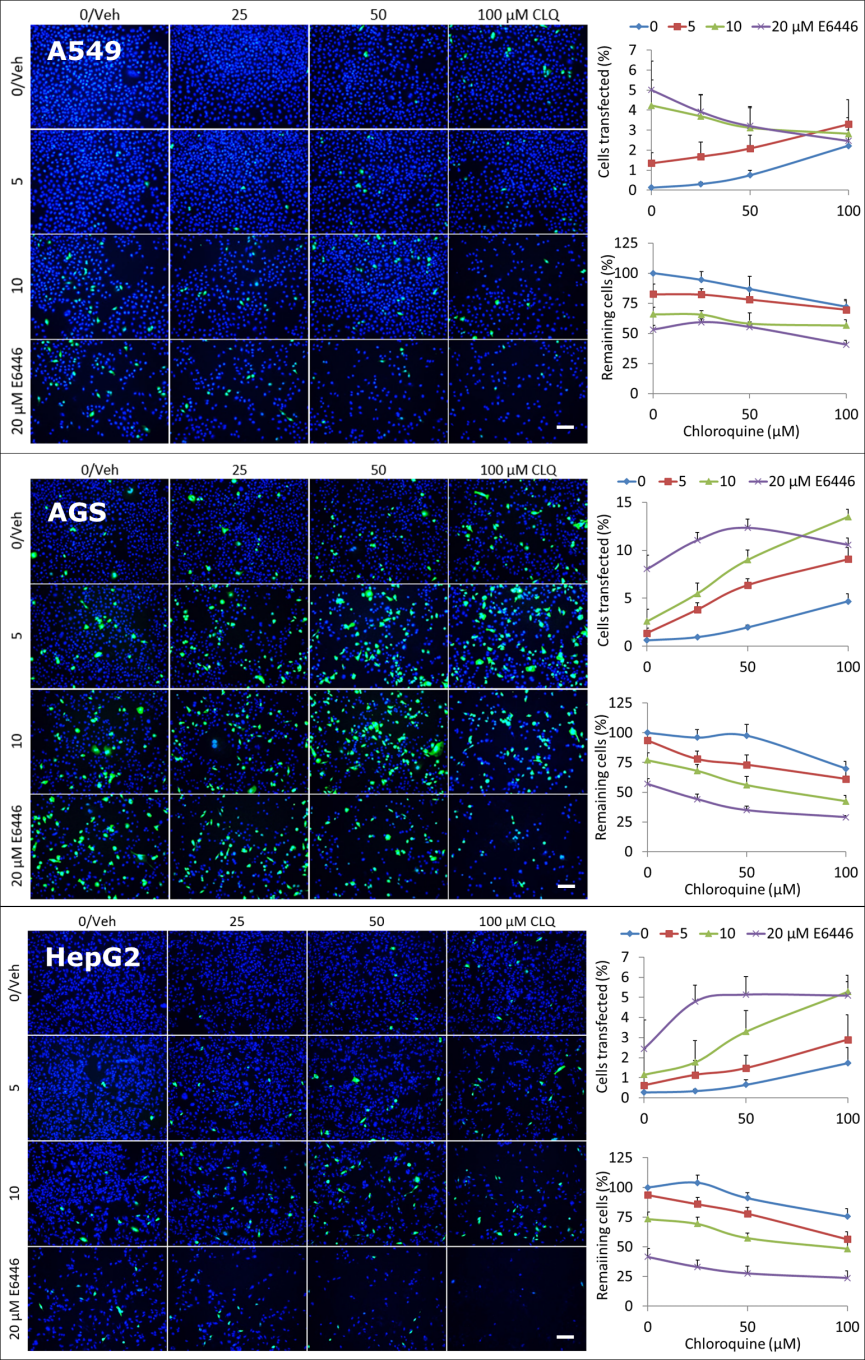
Comparing E6446 and CQ for their ability to enhance XP-mediated transfection of EGFP mRNA. Fluorescence imaging of EGFP expression (green) in A549, AGS, and HepG2 human cancer cell lines 24 h after treatment with EGFP mRNA mixed with XP, E6446 (0–20 μM), and CQ (0–100 μM). Quantified for each cell line and treatment condition are the percentages of cells expressing EGFP (*top graph in each panel*) and the cell counts relative to vehicle-treated controls (*bottom graph in each panel*). Data are shown as mean ± 95% confidence interval and are representative of 4+ independent experiments. Cell nuclei are stained blue with DAPI. Veh = vehicle; scale bars = 100 μm.

To do so, we treated A549, AGS, and HepG2 cells with EGFP mRNA that had been mixed with XP, E6446 (0–20 μM), and CQ (0–100 μM). We found that 5% (A549), 8% (AGS), and 2% (HepG2) of cells expressed EGFP where 20 μM E6446 was added; while 2% (A549), 5% (AGS), and 2% (HepG2) of cells expressed EGFP where 100 μM CQ was added (Fig 2 *images and top graph in each panel*). Thus, E6446 was >5-fold more potent on a concentration basis than CQ at improving EGFP mRNA transfections by XP in these cell lines (S4 Fig). We also found that combining E6446 and CQ at concentrations which on their own produced submaximal EGFP expression additively increased the number of cells expressing EGFP, and that certain combinations improving EGFP expression in AGS and HepG2 cell lines more than E6446 or CQ alone. An example of the latter is the combination of 10 μM E6446 + 100 μM CQ, where 13% of AGS and 5% of HepG2 cells expressed EGFP compared to 8% and 2% expression for 20 μM E6446 and 5% and 2% expression for 100 μM CQ in the same cell lines, respectively. Notably, E6446 and CQ also reduced cell counts in a concentration-dependent manner, with similar reductions across the cell lines for both 10 μM E6446 (23–34%) and 100 μM CQ (25–30%); and combining E6446 with CQ additively reduced cell counts across the cell lines (Fig 2 *bottom graph in each panel*), with the combination of 100 μM CQ + 10 μM E6446 reducing cell counts across the cell lines significantly less than the combination of 100 μM CQ + 20 μM E6446 (2-way ANOVA: F_1,24_ = 17.74; p < 0.001). Finally, it was apparent that at higher E6446 and/or CQ concentrations, such as 20 μM E6446 + 50 or 100 μM CQ, the proportions of cells expressing EGFP decreased less than the overall decreases in cell counts. Thus, while the absolute numbers of cells expressing EGFP decreased under these conditions the percentages of cells expressing EGFP were relatively elevated.

### E6446 enhances transfection of CFTR mRNA by XP

**Fig 3:**
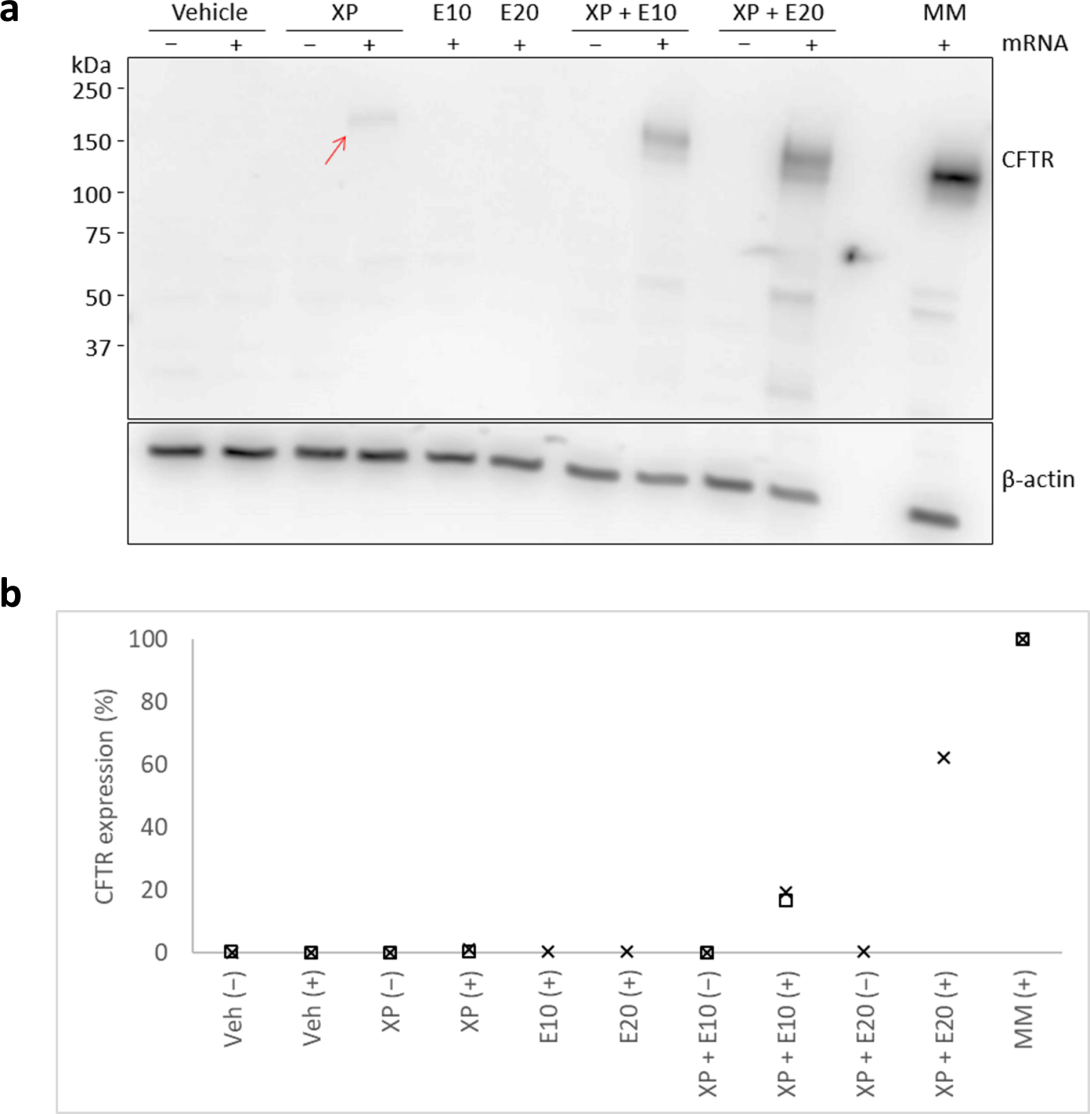
E6446 enhances XP-mediated transfection of CFTR mRNA. Western blot **(a)** and densitometry **(b)** analyses of HEK293T cells lysed 24 h after transfection with 5 μg of modified CFTR mRNA (+) or buffer (−) and 10 μg of XP in the presence or absence of 10 (E10) and/or 20 (E20) μM E6446. A control set of cells was transfected with CFTR mRNA using Lipofectamine™ MessengerMax transfection agent (MM). The blot was probed with antibodies against CFTR and β-actin. The position of the CFTR band (170 kDa) is indicated and an arrow points to a faint CFTR band produced by transfection of CFTR mRNA with XP in the absence of E6446. The densitometry analysis, derived from two independent experiments (⊆,≤), shows data points normalised to β-actin expression and relative to the CFTR expression facilitated by transfection using MessengerMax.

Finally, we investigated the ability of XP and E6446 to transfect mRNA encoding the human CFTR. To do so we cultured 4 x 10^5^ CFTR-deficient human embryonic kidney (HEK293T) cells with 10 μg XP that had been mixed with 5 μg of modified CFTR mRNA and 0, 10, and/or 20 μM E6446. We treated control sets of cells with the mRNA mixed with either vehicle, E6446, or the transfection reagent Lipofectamine^TM^ MessengerMAX (MessengerMAX). The cells were lysed 24 h after treatment and the CFTR expression was examined by Western blotting. We found that transfection by XP produced a CFTR band which was more intense where 10 or 20 μM E6446 was included, albeit CFTR expression did not reach the level obtained by transfection using MessengerMAX (Fig 3A). More specifically, densitometric analysis of the blotting shows that XP, XP + 10 μM E6446, and XP + 20 μM E6446 respectively facilitated approximately 1, 18, and 62% of the CFTR expression facilitated by MessengerMax (Fig 3B).

## Discussion

These results demonstrate that truncated human protamine alone or fused to the CPP Xentry, to form XP, can transfect mRNAs into human cancer cell lines *in vitro*, with up to 2% of cells expressing fluorescent reporter genes following XP transfections. Further, the co-addition of CQ and/or E6446 to reporter gene mRNA transfections by XP increases reporter gene expression to up to 13% of cells, with E6446 being >5-fold more potent than CQ on a concentration basis in enhancing expression. Notably, XP can transfect mRNA encoding the CFTR, with the co-addition of E6446 markedly enhancing CFTR expression. Finally, for reasons not explored here, the addition of E6446 and/or CQ to XP mRNA transfections reduces cell counts in cancer cell lines.

This study is one of the few reporting mRNA transfection by CPPs [36]. This is somewhat surprising given that native fish protamine was observed in 1961 to enhance the uptake of bacterial RNA into cultured cells [37] and that CPPs are well-documented to transfect RNA and DNA oligonucleotides [38–44] and plasmids [30, 45]; though presumably relates to the perceived challenges surrounding the use of mRNA for gene therapy [1]. Interestingly, protamine is used in RNA vaccines [46], though to protect the RNA and to help elicit an immune response rather than to transfect per se [47]. That protamine heightens immune responses to mRNA [48] suggests that using TLR inhibitors, such as E6446 and CQ, or a less immunostimulatory nucleic acid-binding peptide is required when an immune response is not desired.

The modest levels of reporter gene expression observed here following mRNA transfections by XP and E6446 appear to preclude the use of these agents in gene therapy. However, disorders exist where even relatively small increases in gene expression could benefit patients. An example of this is CF, where it has been proposed that a 10% restoration of CFTR activity may be sufficient to alleviate symptoms [49]. Transfection of CFTR mRNA into human cells *in vitro* was first reported by Bangel-Ruland et al. in 2013 [50], though the Lipofectamine^TM^ reagent they used is limited by toxicity and poor transfection *in vivo* [51]. In comparison, CPPs, including Xentry, have shown to be relatively non-toxic in rodents [20, 52, 53]; and whilst the toxicity of XP has not been tested, protamine is widely clinically used as an excipient in neutral protamine Hagedorn (NPH) insulin [54] and to reverse the anticoagulant properties of heparin [55].

The mechanisms by which E6446 enhances mRNA transfections by XP remain to be elucidated, though presumably relate to the suppression of innate immune responses. The additive effects of E6446 and CQ on gene expression, when combined at concentrations that gave submaximal gene expression on their own, suggest that E6446 and CQ enhance transfection by related mechanisms. E6446 along with CQ and HCQ are lipophilic weak bases which accumulate in intracellular acidic compartments where nucleic acid-sensing TLRs 7 and 9 reside. There these reagents bind to nucleic acids and inhibit TLR activation, with E6446 being 5–20 fold more effective at suppressing the activation of TLRs 7 and 9 than CQ [34]. That this difference in potency is consistent with difference between the reagents in enhancing transfections here suggests that suppression of TLR activation may be the mechanism by which E6446 enhances transfection. The use of specific TLR activators and other TLR inhibitors could be useful in understanding E6446’s activity better and may also provide insight into the somewhat differing effects of E6446 on transfection between the cell lines. Notably, however, the optimal E6446 concentrations for assisting mRNA transfections observed here (10–20 μM) greatly exceed the concentrations (IC_50_ 0.1–1.78 μM) previously reported to inactivate single-stranded RNA sensing TLR7 in cell lines [33, 34]. Presumably this discrepancy is related to the immunostimulatory presence of protamine, increasing the E6446 concentrations required to inhibit TLR activation, though this remains to be determined.

Whether XP retains Xentry’s selectivity in only entering adherent cells was not ascertained here, though. Xentry’s specificity relates to an energy-dependent, endocytic cell entry process involving the heparan sulphate side-chains of the syndecan-4 transmembrane receptor proteoglycan [20], a key cell adhesion molecule [56]. In contrast, native fish protamine and low molecular weight protamine are reported to enter many cell types, including non-adherent erythrocytes [57, 58]. In preliminary testing conducted by the authors, though, neither XP nor truncated human protamine transfected mRNA into non-adherent leukaemic (K562 and TK1) cell lines, even with the co-addition of E6446 (data not shown). This finding is consistent with observations that arginine-rich CPPs (such as protamine) lose their ability to enter cells passively, as opposed to receptor-mediated uptake, when attached to large cargoes [22]. As such, truncated human protamine complexed with mRNA may enter adherent cells specifically, which may make Xentry redundant in XP.

Similarly not ascertained, was XP’s ability to target specific tissues through the appendage of a homing peptide and/or a protease-activatable domain. From the literature we had identified a potential lung homing peptide in the form of the amino acid motif CGFECVRQCPERC (GFE-1), which shows tropism towards lung tissue after intravenous injection [59]; and a potential protease-activatable domain in the form of the amino acid sequence KHYR, which is cleavable by membrane-bound prostasin that is highly expressed in lung airways. Thus, fusing GFE-1 and/or the KHYR to XP could be used to target CFTR mRNA to the lung tissue of CF patients. However, preliminary testing *in vitro* of an XP fusion peptide containing the KHYR motif showed non-specific epithelial cell uptake (data not shown), which may be due to the truncated human protamine overriding the activatable domain. As such, tissue-specific targeting using Xentry CPPs may only be achievable by replacing truncated human protamine with a nucleic acid-binding peptide which does not enter cells on its own.

Whether E6446 has utility in mRNA-based therapies requires further investigation. While not tested for toxicity in humans, E6446 has proven to be relatively non-toxic in mice [33–35]. The highest dose of E6446 reported in testing, 120 mg/kg per day given orally for 12 days, produced a serum concentration of 1 μM. Notably, a 60 mg/kg dose completely inhibited IL-6 production in sera 2 h after challenge with oligonucleotide-containing CpG motifs, which is to be expected given the low IC_50_ for TLR9 inhibition [34]. Whilst a serum concentration of 1 μM is 10-20-fold less than what was observed to be optimal to enhance mRNA transfection in the present study, Franklin et al. reported that this level of E6446 protected malaria-infected mice from TLR4-mediated LPS-induced shock. The authors argued that it is conceivable, therefore, that E6446 administered at 120 mg/kg could inhibit the *in vivo* activation of TLR7/8, which has a lower IC_50_ than inhibition of TLR4 [33]. Thus, testing E6446 *in vivo* for utility in enhancing mRNA therapy delivery could be warranted.

Finally, whether or not E6446 proves suitable for use in gene therapy, the current study suggests that E6446 may have anticancer activity like CQ. CQ is being tested as a cancer therapeutic, as neoplastic cells are known to be more sensitive to CQ than their non-transformed counterparts [60]. The anticancer activity of CQ is thought due to autophagy and signal transduction inhibition and apoptosis initiation via lysosomal membrane permeabilization [60]. Thus, the reduced cancer cell counts associated with CQ treatment observed here are presumably due to cell death. Considering the similarities between CQ and E6446, the reduced cancer cell counts associated with E6446 observed here were presumably also due to cell death. This, however, remains to be determined. Toxicity assays performed on cancer cells lines, non-tumour cell lines, and primary cells would determine this and whether E6446 has a selective toxic effect on cancer cells. If it does, then the ability of E6446 to kill carcinoma cells 5-10-fold more potently than CQ would indicate anticancer activity worth exploring further.

In summary, the current study demonstrates the feasibility of mRNA transfection by protamine-based CPPs potentiated by E6446. Potentially this approach could be utilized for mRNA-based gene therapy to treat disorders, such as CF, where modest restorations in gene activity may have a meaningful impact on disease progression. Whilst this prospect may be some way off, the findings described here could be of immediate practical relevance to researchers seeking to improve the efficiency of mRNA transfection by their preferred CPP.

## Materials and methods

### Cell lines

Cell lines were obtained from the American Type Culture Collection (Manassas, USA). They were cultured in Ham's F12 Nutrient Mixture (AGS and A549 cells), DMEM/F12 (HepG2, HT29, and MCF-7 cells), and DMEM (HEK293T cells) media supplemented with 10% FBS (all supplied by Thermo Fisher Scientific, Waltham, USA) and incubated at 37°C with 5% CO_2_.

### Transfection reagents

Biotinylated D-isomeric XP (amino acid sequence: lclrpvggrsqsrsryyrqrqrsrrrrrrs) and FITC-labelled protamine (amino acid sequence: rsqsrsryyrqrqrsrrrrrrs) and XP were synthesized by Peptide 2.0 (Chantilly, USA), dissolved in water to 0.5 mM, and stored as single-use aliquots at −80°C. E6446 (Merck Millipore, Molsheim, France) was dissolved to 20 mM in DMSO (Sigma-Aldrich, Auckland, NZ), chloroquine diphosphate (Sigma-Aldrich, St. Louis, USA) and hydroxychloroquine sulfate (Sigma, St. Louis, USA) were dissolved to 30 mM in water, and then these reagents were aliquotted and stored at −20°C.

### RFP and EGFP mRNA transfection

Cells (1 × 10^4^/well) were seeded in flat-bottomed, tissue culture-treated 96-well plates (BD Biosciences, Auckland, NZ) and cultured overnight. XP and mRNAs (mCherry RFP and EGFP; TriLink Biotechnologies, San Diego, USA) were diluted in Opti-MEM I Reduced Serum Medium (Thermo Fisher Scientific, Waltham, USA) then mixed (1 μg XP:0.25 μg mRNA) and placed on a rocker at room temperature in the dark for 15 min. The solutions were then mixed with vehicle, E6446, CQ, and/or HCQ that had been diluted in cell culture media and then 100 μL of each mixture was added to the cells. The cells were then cultured for 4 h whereupon the treatment solutions were replaced with normal media and cultured for 20 h. Quantified experiments were repeated independently at least four times.

### Fluorescence microscopy and quantitation

Twenty-four hours after treatment, cells were either nuclear counterstained with NucBlue™ (Molecular Probes, Eugene, USA) or fixed with 4% formaldehyde (diluted in PBS) and counterstained with DAPI (Molecular Probes, Eugene, USA). Epifluorescence images (100 × magnification) were recorded using a Nikon (Tokyo, Japan) Eclipse TE2000-S microscope, Digital Sight DS-U1 PC controlled colour camera, and NIS-Elements F software. Where quantification was involved the images were taken at 5+ non-overlapping areas per well and reporter gene expression was quantified using ImageJ software [61] and custom macros.

### Preparation of CFTR mRNA

A pcDNA3.1 plasmid encoding the human CFTR (NM_000492.3) was obtained from GenScript (OHu27239; Piscataway, USA). The plasmid (100 ng) was transformed into E. cloni 10G Chemically Competent Cells (Lucigen, Middleton, USA), cultured on LB agar with ampicillin (100 μg/mL) for 2 days, selected, and amplified in LB-Miller medium with 100 μg/mL ampicillin (at 28°C, 200 rpm) for 2 days. The amplified plasmid was isolated using a NucleoBond Xtra Midi Kit (Macherey-Nagel, Düren, DE) and then linearized to generate a DNA template for *in vitro* transcription. More specifically, 10 μg plasmid was incubated with 175 U of Not I-HF restriction endonuclease (New England Biolabs, Ipswich, USA) for 4 h at 37°C. The digest was then separated on a 0.7% gel prepared using Agarose LE (Roche, Mannheim, DE) and TAE buffer (10 mM Tris-Cl (pH 8.0), 1 mM EDTA (pH 8.0)). After separation the DNA was stained with SYBR Safe dye (Invitrogen, Carlsbad, USA), excised from the gel using a scalpel and a Safe Imager Blue Light Transilluminator (Thermo Scientific, Carlsbad, USA), purified using a PureLink Quick Gel Extraction Kit (Invitrogen, Carlsbad, USA), incubated with Proteinase K (100 μg/mL; New England Biolabs, Ipswich, USA) and SDS (0.5%) at 50˚C for 30 min, extracted with phenol (Sigma-Aldrich, Auckland, NZ)/chloroform (EMD Millipore, Billerica, USA), and precipitated with ethanol.

The linear template was transcribed *in vitro* to cap-1, Ψ-containing CFTR mRNA using CellScript (Madison, USA) products: INCOGNITO T7 Ψ-RNA Transcription Kit, ScriptCap m7G Capping System, and the ScriptCap 2'-O-Methyltransferase Kit; and the mRNA was polyadenylated using the CellScript A-Plus Poly(A) Polymerase Tailing Kit, including a 90 min incubation to generate 200+ base tails. The mRNA was recovered using Lithium Chloride Precipitation Solution (Thermo Fisher Scientific, Vilnius, Lithuania), quantified by Nanodrop 2000 (Thermo Fisher Scientific, Waltham, USA), analysed via bleach agarose gel electrophoresis [62], and stored in nuclease-free water at −80°C.

### Transfection of cells with CFTR mRNA

Cells were seeded at 4 x 10^5^ cells/well in tissue culture-treated 12-well plates (BD Biosciences, Auckland, NZ) in 1 mL of culture media and cultured overnight. Transfection reagents, XP (10 μg) and Lipofectamine™ MessengerMax (3 μL; Thermo Fisher Scientific, Carlsbad, USA), and CFTR mRNA (5 μg) were separately diluted in Opti-MEM I Reduced Serum Medium to 50 μL volumes and incubated for 5–10 min at room temperature. Transfection reagent and mRNA solutions were then combined as appropriate and incubated at room temperature for 5 min. Vehicle/DMSO and E6446 were then mixed into these solutions as indicated, the solutions added dropwise to the media in the appropriate wells, and the plates rocked gently by hand to disperse the treatment solutions. The cells were then cultured for 24 h, with the treatment solutions replaced with normal culture medium after 4 h.

Statistical analyses, in the form of two-way analysis of variance (ANOVA), were performed using IBM SPSS Statistics (version 19; Chicago, USA); with the significance level set at or below 5%. Standard error of the mean and confidence intervals were calculated using Microsoft Excel 2010.

### Western blot analysis of cells transfected with CFTR mRNA

Cells were washed with 1 mL of fridge-cold PBS, lysed with 50 μL of 1% Triton X-100 lysis buffer (1 mM Tris-HCl, 15 mM NaCl, 0.2 mM EDTA, 1% Triton X-100, and 1x protease inhibitor (Sigma-Aldrich, Auckland, NZ)), and placed on ice on a rocker for 30 min. The lysates were then collected by cell scraper and centrifuged at 13,000 g for 15 min at 4°C, whereupon the supernatants were collected and their protein concentrations measured using a bicinchoninic acid (BCA) assay (Thermo Fisher Scientific, Waltham, USA). Samples containing ~7–20 μg of lysate protein were then mixed with 4x SDS sample buffer (250 μM Tris-HCl (pH 6.8), 8% SDS, 40% glycerol, 200 mM DTT, and bromophenol blue), incubated at 37°C for 15 min, and then resolved on 6% (w/v) polyacrylamide Tris-tricine SDS gels. A semi-dry Hoeffer TE77 unit (GE Healthcare, Uppsala, Sweden) was used (50 mA for 1.5 h) to transfer proteins to PVDF membranes (GE Healthcare, Uppsala, Sweden). Blots were then blocked with 5% non-fat dry milk in Tris-buffered saline/Tween (TBST; 10 mM Tris HCl (pH 7.4), 140 mM NaCl, 0.1% Tween 20) for 1 h at room temperature and then incubated overnight at 4°C with a mouse monoclonal anti-CFTR antibody (MM13.4; Merck Millipore, Darmstadt, Germany) diluted at 1:1,000 in 4 mL of 5% BSA/TBST, as described previously [63]. The blots were then washed with TBST and incubated with goat anti-mouse (A90-105P; Bethyl, Montgomery, USA) IgG conjugated with horseradish peroxidase and diluted 1:10,000 in TBST containing 5% non-fat dry milk for 1 h at room temperature. The blots were then washed again with TBST and the antigens detected using ECL Advance (GE Healthcare, Uppsala, Sweden) and a Fujifilm LAS-3000 CCD camera-based luminescence imager (Tokyo, Japan). The blots were subsequently reprobed with a rabbit polyclonal anti-β actin antibody (AB8227, 1:10000; Abcam, Cambridge, USA) to detect β-actin as a loading control. Densitometric analysis of the Western blots was undertaken using ImageJ as described previously [64].

## Supporting information

S1 Fig XP transfects mRNA at low levels into human cancer cells. Epifluorescence microscopy images of EGFP expression (green) in live AGS cells 24 h after treatment with XP which had been mixed with EGFP mRNA at XP:mRNA (w/w) ratios ranging from 2:1 to 16:1. As controls, other sets of cells were treated with EGFP mRNA only or mRNA mixed with Lipofectamine™ 2000 (L2000) transfection agent. The cells were nuclear counterstained with NucBlue™ (blue); scale bar = 100 μm.
(TIFF)

S2 Fig Truncated human protamine and XP transfect mRNA at low levels into human cancer cells. Epifluorescence microscopy images of RFP expression (red) in live AGS cells 24 h after treatment with RFP mRNA mixed with either truncated human protamine or XP. As a control, another set of cells were treated with RFP mRNA mixed with Lipofectamine™ 2000 (L2000) transfection agent. The cells were nuclear counterstained with NucBlue™ (blue) prior to imaging, and the RFP and nuclear staining images are shown here merged (scale bar = 100 μm).
(TIFF)

S3 Fig Chloroquine (CQ) and hydroxychloroquine (HCQ) enhance mRNA transfection by XP. Epifluorescence microscopy images of EGFP expression (green) in AGS cells 24 h after treatment with EGFP mRNA mixed with XP and either CQ or HCQ (0–100 μM). As controls, other sets of cells were treated with EGFP mRNA (in the absence of XP) to which 100 μM CQ or HCQ had been added. The fixed cells were nuclear counterstained with DAPI (blue); scale bar = 100 μm.
(TIFF)

S4 Fig E6446 is >5-fold more potent than CQ at improving EGFP mRNA transfection by XP. A plot showing the percentages of A549, AGS, and HepG2 cells expressing EGFP 24 h after transfection of EGFP mRNA using XP and E6446 (5–20 μM) or chloroquine (25–100 μM). Data are representative of 4+ independent experiments and the standard errors of the means (SEM) are shown.
(TIFF)
