# Supplementary figures and images for: mRNA transfection by a Xentry-protamine cell-penetrating peptide is enhanced by TLR antagonist E6446
Source: PLoS One. 2018 Jul 30;13(7):e0201464. doi: 10.1371/journal.pone.0201464 (PMC6066245; doi:10.1371/journal.pone.0201464)

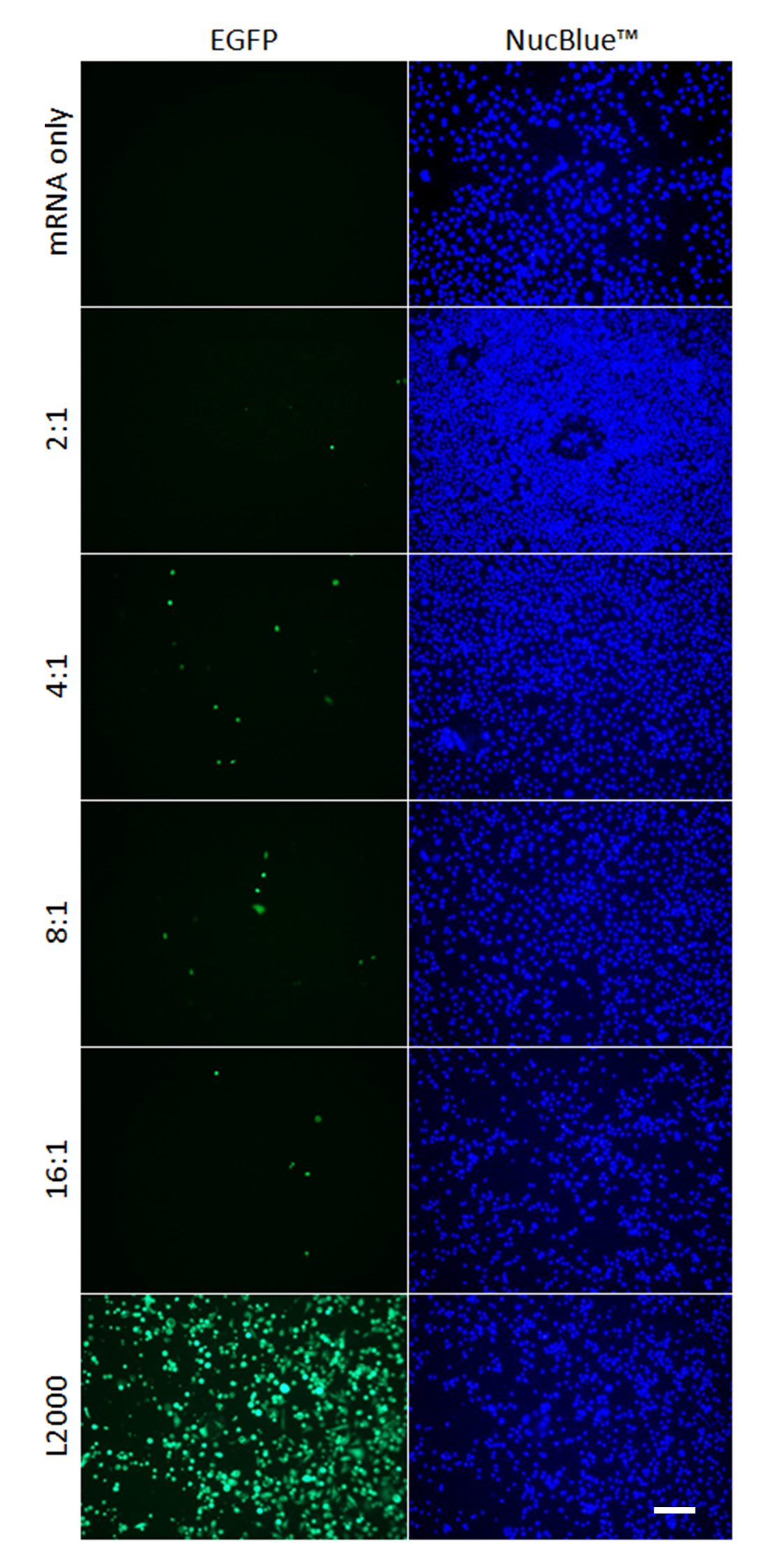

Supplement: S1 Fig — Epifluorescence microscopy images of EGFP expression (green) in live AGS cells 24 h after treatment with XP which had been mixed with EGFP mRNA at XP:mRNA (w/w) ratios ranging from 2:1 to 16:1. As controls, other sets of cells were treated with EGFP mRNA only or mRNA mixed with Lipofectamine™ 2000 (L2000) transfection agent. The cells were nuclear counterstained with NucBlue™ (blue); scale bar = 100 μm. (TIFF) [file pone.0201464.s001.tiff]

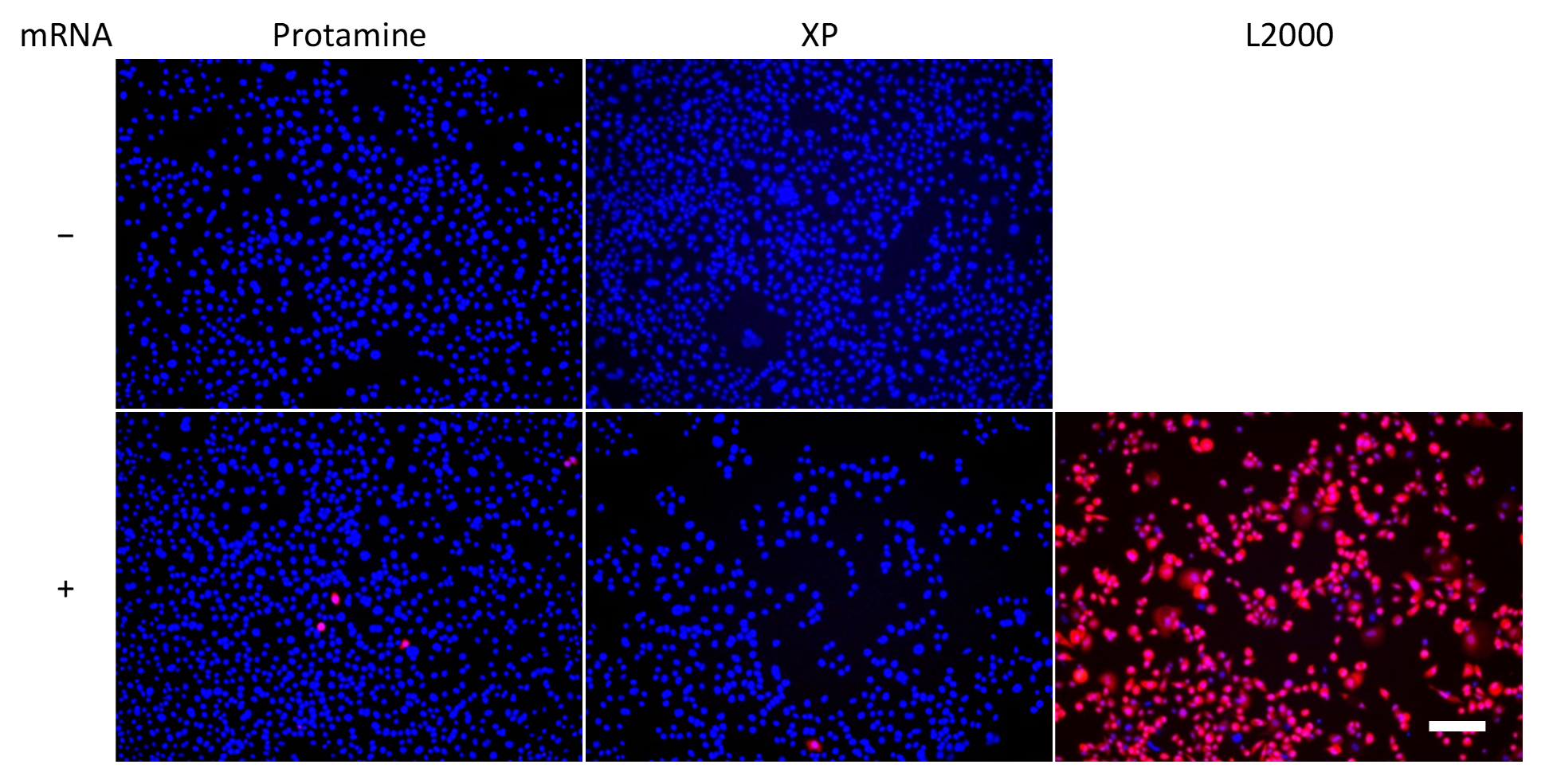

Supplement: S2 Fig — Epifluorescence microscopy images of RFP expression (red) in live AGS cells 24 h after treatment with RFP mRNA mixed with either truncated human protamine or XP. As a control, another set of cells were treated with RFP mRNA mixed with Lipofectamine™ 2000 (L2000) transfection agent. The cells were nuclear counterstained with NucBlue™ (blue) prior to imaging, and the RFP and nuclear staining images are shown here merged (scale bar = 100 μm). (TIFF) [file pone.0201464.s002.tiff]

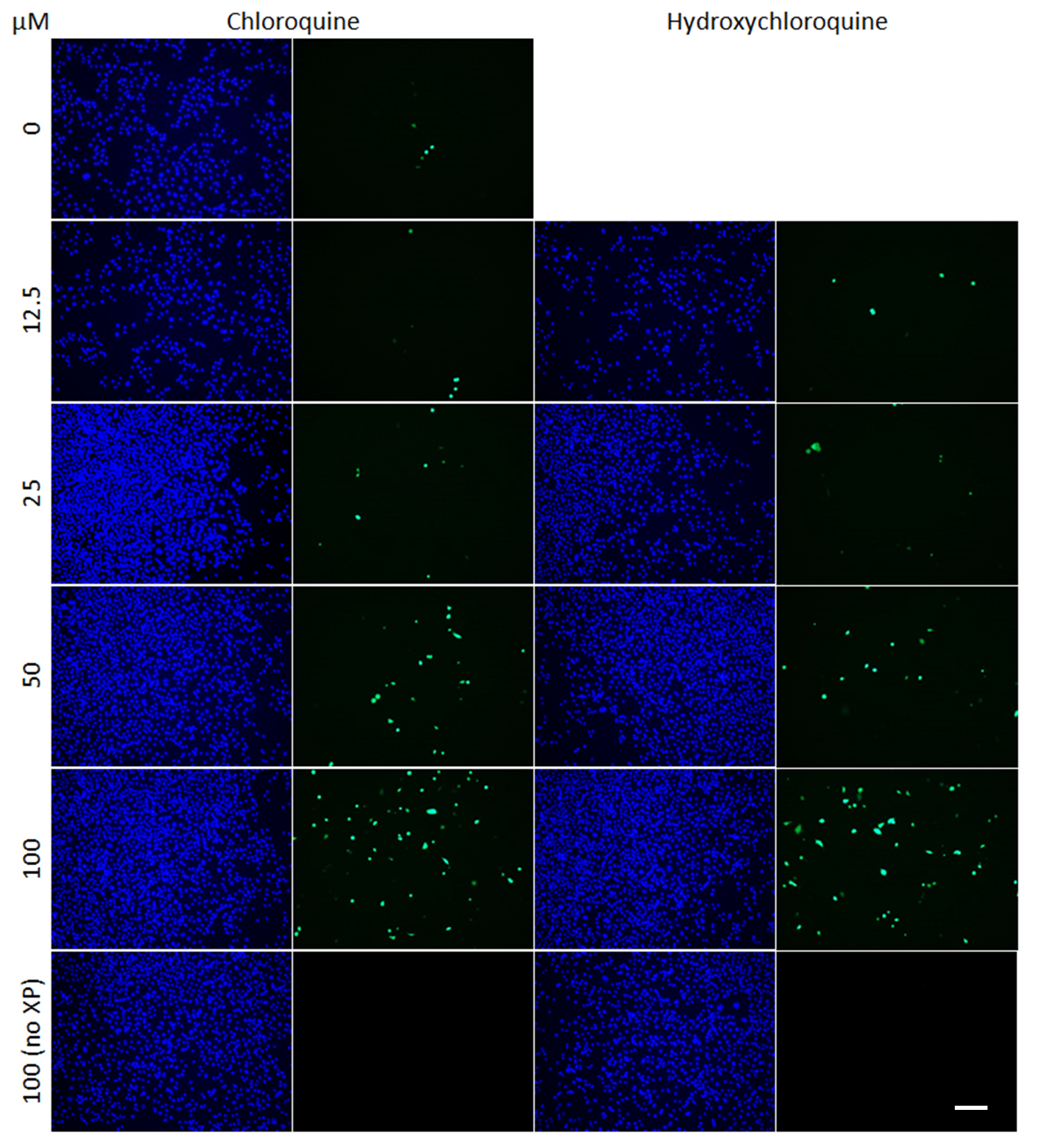

Supplement: S3 Fig — Epifluorescence microscopy images of EGFP expression (green) in AGS cells 24 h after treatment with EGFP mRNA mixed with XP and either CQ or HCQ (0–100 μM). As controls, other sets of cells were treated with EGFP mRNA (in the absence of XP) to which 100 μM CQ or HCQ had been added. The fixed cells were nuclear counterstained with DAPI (blue); scale bar = 100 μm. (TIFF) [file pone.0201464.s003.tiff]

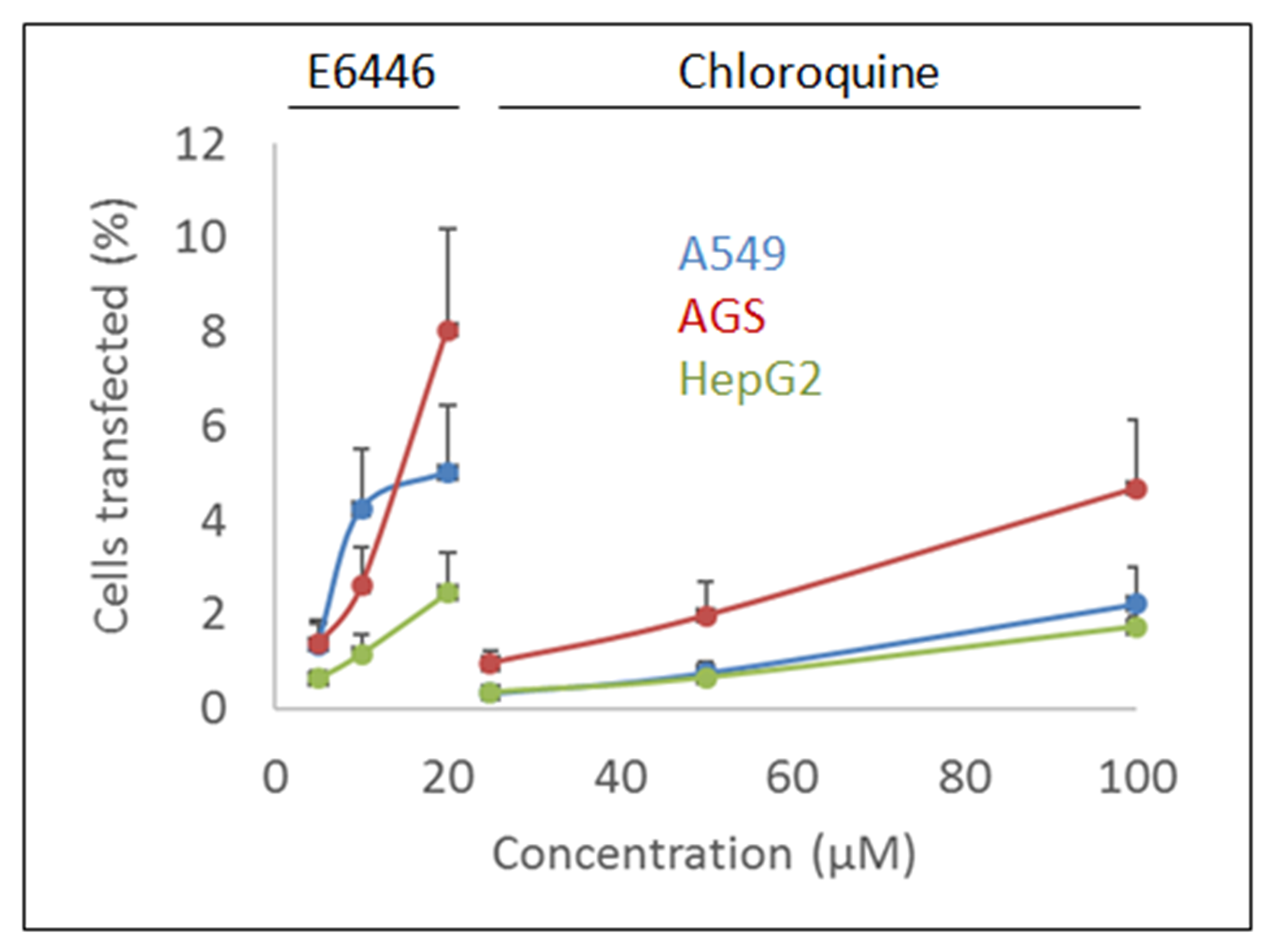

Supplement: S4 Fig — A plot showing the percentages of A549, AGS, and HepG2 cells expressing EGFP 24 h after transfection of EGFP mRNA using XP and E6446 (5–20 μM) or chloroquine (25–100 μM). Data are representative of 4+ independent experiments and the standard errors of the means (SEM) are shown. (TIFF) [file pone.0201464.s004.tiff]
